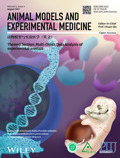# Cover Picture

**DOI:** 10.1002/ame2.12244

**Published:** 2023-09-08

## Abstract

The cover image is based on the article ‘Transcriptomic and proteomic studies of condylar ossification of the temporomandibular joint in porcine embryos’(Doi: 10.1002/ame2.12326) reported by Lei Xiang, Yongfeng Li, et.al. Transcriptome and proteomic analysis during the embryonic development of temporomandibular joint in miniature pigs revealed the key regulatory genes and proteins for condylar ossification during embryonic development. This research finding could provide a basis for the pathogenesis of temporomandibular joint, tissue engineering, and regenerative medicine.